# Supplementary material for: Empirical Myoelectric Feature Extraction and Pattern Recognition in Hemiplegic Distal Movement Decoding
Source: Bioengineering (Basel). 2023 Jul 21;10(7):866. doi: 10.3390/bioengineering10070866 (PMC10376258; doi:10.3390/bioengineering10070866)
Supplement: Supplementary file 1 [file bioengineering-10-00866-s001.zip › bioengineering-2417524-supplementary.pdf]

Supplementary Materials

Empirical Myoelectric Feature Extraction and Pattern Recognition in Hemiplegic Distal Movement Decoding

Alexey Anastasiev [1], Hideki Kadone [2,*], Aiki Marushima [3], Hiroki Watanabe [3], Alexander Zaboronok [3], Shinya Watanabe [3], Akira Matsumura [4], Kenji Suzuki [5], Yuji Matsumaru [3] and Eiichi Ishikawa [3]

[1] Department of Neurosurgery, Graduate School of Comprehensive Human Sciences, University of Tsukuba, 1-1-1 Tennodai, Tsukuba 305-8575, Ibaraki, Japan; s1936046@s.tsukuba.ac.jp
[2] Center for Cybernics Research, Institute of Medicine, University of Tsukuba, 1-1-1 Tennodai, Tsukuba 305-8575, Ibaraki, Japan
[3] Department of Neurosurgery, Institute of Medicine, University of Tsukuba, Tennodai 1-1-1, Tsukuba 305-8575, Ibaraki, Japan; aiki.marushima@md.tsukuba.ac.jp (A.M.); watanabe.hiroki.gb@u.tsukuba.ac.jp (H.W.); a.zaboronok@md.tsukuba.ac.jp (A.Z.); shinya-watanabey@md.tsukuba.ac.jp (S.W.); yujimatsumaru@md.tsukuba.ac.jp (Y.M.); e-ishikawa@md.tsukuba.ac.jp (E.I.)
[4] Ibaraki Prefectural University of Health Sciences, 4669-2 Amicho, Inashiki 300-0394, Ibaraki, Japan; matsumura.akira.ft@alumni.tsukuba.ac.jp
[5] Center for Cybernics Research, Artificial Intelligence Laboratory, Faculty of Engineering Information and Systems, University of Tsukuba, 1-1-1 Tennodai, Tsukuba 305-8573, Ibaraki, Japan; kenji@ieee.org
* Correspondence: kadone@ccr.tsukuba.ac.jp

Supplementary sources

Table S1. Universal feature collector of time domain (TD), frequency domain (FD), time–frequency domain (TFD), fractal domain (FRD), and spatial domain (SD) features. .

Feature	Abbreviation	Parameters	Short description
**IEMG**	Integrated EMG parameter	$\mathrm{IEMG} = \sum_{n=1}^{N} \lvert x_n \rvert$	IEMG shows the numerical summation of the absolute values of the EMG signals amplitude (point by point), which is used for motor unit firing sequences detection.
**AAV**	Average amplitude value	$\mathrm{AAV} = \frac{1}{N}\sum_{n=1}^{N} x_n$	AAV is the basic time domain feature that calculates the average amplitude of the myoelectrical signal.
**MAV**	Mean absolute value	$\mathrm{MAV} = \frac{1}{N}\sum_{n=1}^{N} \lvert x_n \rvert$	MAV is a generalized parameter of muscle contraction that reflects the average absolute value of the EMG signal's amplitude. MAV is primarily used to track muscle activity onset. As an elementary function, MAV is flexible for wide-range modifications thereby being ranked as the main feature in pattern recognition.
**LMAV**	Log of the mean absolute value	$\mathrm{LMAV} = \log_e(\mathrm{MAV})$	LMAV simulates the nonlinear scaling shape of the original MAV function. The feature enhances the window discrimination and orients on the low amplitude of the EMG signal.
**MMAV1**	Modified mean absolute value type 1	$\mathrm{MMAV1} = \frac{1}{N}\sum_{n=1}^{N} w_n \lvert x_n \rvert$ $w_n = \begin{cases} 1, \text{if } 0.25N \le n \le 0.75N \\ 0.5, \quad \text{otherwise} \end{cases}$	MMAV1 expands MAV potential using the weighting window function (w_n) of a given frame signal segment in order to enhance pattern recognition.
**MMAV2**	Modified mean absolute value type 2	$w_n = \begin{cases} 1, \text{if } 0.25N \le n \le 0.75N \\ \frac{4n}{N}, \text{ if } n < 0.25N \\ \frac{4(n-N)}{N}, \text{otherwise} \end{cases}$	MMAV2 operates similarly to MMAV1; however, the feature implements the continuous weighted window function.
**MMAV3**	Modified mean absolute value type 3	$w_n = \frac{n}{N}$	Additionally, the continuous range can be further modulated by specific window settings (MMAV3-MMAV6), which we have crafted during EMG acute stroke data decoding.
**MMAV4**	Modified mean absolute value type 4	$w_n = \begin{cases} \frac{N}{n} \\ 1 - \frac{n}{N} \end{cases}$	
**MMAV5**	Modified mean absolute value type 5	$w_n = \begin{cases} \frac{4n}{N}, n < 0.25N \\ \frac{4n}{N} - 1, \ 0.25N \le n \le 0.5N \\ \frac{4n}{N} - 2, 0.5N \le n \le 0.75N \\ \frac{4n}{N} - 3, \text{otherwise} \end{cases}$	

**MMAV6**	Modified mean absolute value type 6	$w_n = \begin{cases} n < \frac{3}{8}N \\ \frac{3}{8}N < n < \frac{5}{8}N \\ n > \frac{5}{8}N, \text{otherwise} \end{cases}$	
**MAVS**	Mean absolute value slope	$MAVS_i = MAV_{i+1} - MAV_i$<br>$i = 1, \ldots, I-1$	MAVS calculates the difference in a set of adjacent MAV segments, whereas the number of segments (I) can be specified depending on the intended signal representation.
**EMAV**	Enhanced mean absolute value	$EMAV = \frac{1}{N}\sum_{n=1}^{N}\lvert(x_n)^p\rvert$<br>$p = \begin{cases} 0{,}75, \text{if} \geq 0.2N \,\&\, n \leq 0.8N \\ 0.5, \text{otherwise} \end{cases}$	EMAV is a MAV temporal modification that enhances the feature performance due to the translation of EMG signal length content onto the middle range of the segment. p is the parameter of enhanced mean absolute value.
**SSI**	Simple square integral	$SSI = \sum_{n=1}^{N}\lvert x_n\rvert^2$	SSI is the sum of squared parameters of signal amplitude that imprints the EMG signal energy.
**LSSI**	Log of the simple square integral	$LSSI = \log\sum_{n=1}^{N}\lvert x_n\rvert^2$	LSSI evaluates SSI (EMG signal energy) in the logarithmic scale to highlight weak low-amplitude components of the signal.
**HPA, VAR**	Hjorth parameter activity; variance of the EMG	$HPA = VAR(x) = \frac{1}{N-1}\sum_{n-1}^{N}{x_n}^2$	VAR is a power index of the EMG signal that monitors muscle contracture.<br>HPA (or VAR) is the variance of the EMG signal and represents the total energy of the signal.
**LVAR**	Log of variance	$LVAR = \log(VAR)$	LVAR is a non-linear transformation of the VAR feature.
**HPM**	Hjorth parameter mobility	$HPM = \sqrt{\frac{VAR\left(\frac{dx(n)}{dn}\right)}{VAR(x_n)}}$	HPM is the standard deviation of the derivative of the signal, normalized by the variance of the signal (HPA). This feature measures the rate of change of the EMG signal.
**HPC**	Hjorth parameter complexity	$HPC = \sqrt{\frac{HPM\left(\frac{dx(n)}{dn}\right)}{HPM(x_n)}}$	The third dimensionless Hjorth's parameter (HPC) is the standard deviation of the derivative of the mobility, normalized by the mobility (HPM). HPC measures the rate of change of the given EMG signal length and gives an indication of the complexity of the signal (similar to a pure sine wave).
**TM3-5**	Absolute value of 3rd, 4th, and 5th temporal moments	$TM_m = \left\lvert\frac{1}{N}\sum_{n=1}^{N}x_n^m\right\rvert$, m=3, 4, 5	TM3, TM4, and TM5 represent the high-order statistical baseline for classification.
**MSKEW1**	Modified skewness type 1	$MSKEW1 = \frac{N}{(N-1)(N-2)\sigma^3}\sum_{n=1}^{N}(x_n-\mu)^3$	MSKEW1 is the simple skewness based on quartile evaluation. This feature measures the asymmetry of the amplitude of the EMG signal which represents normal Gaussian distribution. μ is the mean (average) of the distribution of the given signal; σ is the standard deviation.
**MSKEW2**	Modified skewness type 2	$MSKEW2 = \frac{\mu - q_{0.5}}{E[\lvert x-\mu\rvert]}$	MSKEW2 is a skewness based on mean-median difference, standardized by absolute deviation.<br>E represents the expected value or the mean of the cumulative distribution function of the data.

**MSKEW3**	Modified skewness type 3	$\mathrm{MSKEW3} = \frac{\mu - q_{0.5}}{\sigma}$	MSKEW3 is a skewness based on mean-median difference, standardized by standard deviation. $q_{0.5}$ is the interquartile range of the dispersion of the 50th percentile or the median, σ is the standard deviation.
**KURT**	Kurtosis	$\mathrm{KURT} = \frac{M_4}{(M_2)^2}$ $M_k = \frac{1}{N}\sum_{n=1}^{N}(x_n - x)^k$	KURT provides a measurement of isometric muscle contractions that do not depend on EMG signal amplitude. In the scope of signal processing, KURT identifies the statistical distribution shape of a signal versus a normal Gaussian distribution.
**MKURT**	Modified kurtosis	$\mathrm{MKURT} = \frac{(q_{0.875} - q_{0.625}) + (q_{0.375} - q_{0.125})}{q_{0.75} - q_{0.25}}$	MKURT is the octile-based measurement of kurtosis.
**RMSV2-3**	The value of root mean square of 2nd and 3rd order	$\mathrm{RMSV}\gamma = \left(\frac{1}{N}\sum_{n=1}^{N} x_n^{\gamma}\right)^{\frac{1}{\gamma}}$	In the prism of signal processing, RMS is a Gaussian random process within certain amplitude modulation that shows constant muscle force or its synchronization during certain activity.
**LRMSV2-3**	Log of the 2nd and 3rd order root mean square	$\mathrm{LRMSV}\gamma = \log\left(\frac{1}{N}\sum_{n=1}^{N} x_n^{\gamma}\right)^{\frac{1}{\gamma}}$	The logarithmic conversion of the RMS is able to deviate the feature-specific difference thereby is able to enhance the prediction of certain muscle events.
**RSM0**	Root squared zero order moment	$\mathrm{RSM}_0 = \sqrt{\sum_{n=1}^{N-1} x_n^2}$	Analogically to RMS, root squared zero order moment estimates the total signal power which reflects the strength of certain muscle contractions.
**RSD1**	First-order root squared normalized descriptor	$\mathrm{RSD1} = \frac{1}{N}\sum_{n=0}^{N-1} Dx_1[n]^2$	RSD1 is the first difference in approximate derivatives Dx_1 of the EMG signal in every window obtained from the value difference of adjacent elements x_n.
**RSD2**	Second-order squared normalized descriptor	$\mathrm{RSD2} = \frac{1}{N}\sum_{n=0}^{N-1} Dx_2[n]^2$	RSD2 uses second difference derivates Dx_2, but the same calculation procedure as RSD1. Both RSD1 and RSD2 features measure the spectral information of the muscle force from the EMG.
**ASR**	The absolute value of the summation of square root	$\mathrm{ASR} = \left\lvert\sum_{n=1}^{N}(x_n)^{\frac{1}{2}}\right\rvert$	ASR is the integral of the rectified EMG signal, which is supposed to be retaining the entire energy characteristics of the given signal.
**MSR**	The mean value of square root	$\mathrm{MSR} = \frac{1}{N}\sum_{n=1}^{N}(\lvert x_n\rvert)^{\frac{1}{2}}$	MSR estimates the total amount of myoelectrical activity per window of the EMG signal.
**ASM**	The absolute value of the summation of the expth root of the signal data and its mean	$\mathrm{ASM} = \left\lvert\frac{\sum_{n=1}^{N}(x_n)^a}{N}\right\rvert$ $a = \begin{cases} 0.5, \text{if } (n \geq 0.25N \text{ and } n \leq 0.75) \\ 0.75, \text{otherwise} \end{cases}$	ASM measures the approximate signal's amplitude of the given EMG waveform that returns the rectified sum conformation of muscle burst per unit of time. In mathematical definition, ASM is a modification of RSMV2 and WL in which the exp variable is predefined within two possible meanings of 0.5 or 0.75 depending on the characteristics of signal data.

**MANC**	Mean absolute Napier's constant value	$\mathrm{MANC} = \left\lvert \frac{1}{N}\sum_{n=1}^{N} x_n^e \right\rvert$	An EMG signal with a higher value of MANC is more complex, while a lower value indicates simplicity.
**SD**	Standard deviation	$\mathrm{SD} = \sqrt{\frac{1}{N}\sum_{n=1}(x_n - \bar{x})^2}$	SD similarly to RMS is defined as a value of the square root of the variance. In which x is the average amplitude value of the signal.
**LOG**	Log detector	$\mathrm{LOG} = e^{\frac{1}{N}\sum_{n-1}^{N}\log(\lvert x_n\rvert)}$	Non-linear detector LOG estimates the grade of muscle contraction.
**ROG**	Root mean squared normalized value of the log detector	$\mathrm{ROG} = \sqrt{\frac{1}{N} e^{\frac{1}{N}\sum_{n-1}^{N}\log(\lvert x_n\rvert)}}$	ROG similar to LOG is a nonlinear feature that is focused on the estimation of total muscular contraction per window unit at the absolute scale to enlarge the scope of the myoelectrical content.
**MDV**	Median differential value	$\mathrm{MDV} = \mathrm{median}(\lvert x_{n+1} - x_n\rvert)$	MDV is the difference between the median of the signal and the median of the first difference of the EMG signal
**MPD**	Median power difference value	$\mathrm{MPD} = \mathrm{median}(\lvert x_{n+1}\rvert - \lvert x_n\rvert)^2$	MPD estimates the Gaussian noise passage in the nearby continuous signal data.
**DASDV**	Difference absolute standard deviation value	$\mathrm{DASDV} = \sqrt{\frac{1}{N-1}\sum_{n=1}^{N-1}(x_{n+1} - x_n)^2}$	DASDV is a standard deviation of the WL feature that is likewise waveform indicates the complexity of the myoelectrical signal.
**MFL**	Maximum fractal length	$\mathrm{MFL} = \log_{10}\sqrt{\sum_{n=1}^{N-1}\left(x(n+1) - x(n)\right)^2}$	MFL is a logarithmic feature that analyzes the EMG signal fractal length. MFL is used as a navigation feature for tracking low-grade bound muscle activity.
**WL**	Waveform length	$\mathrm{WL} = \sum_{n=1}^{N-1}\lvert x_{n+1} - x_n\rvert$	WL is the complexity parameter aggregating the length, amplitude, and frequency of the given signal's time-series segment. WL is the elongation of the IEMG parameter that shows the cumulative span of the waveform over the segment.
**WLR**	Waveform length ratio	$\mathrm{WLR} = \log\left(\frac{\sum_{n=1}^{N}\lvert x_{n+1} - x_{n-1}\rvert}{\sum_{n=1}^{N}\lvert x_{n+2} - x_{n+2}\rvert}\right)$	WLR is the WL modification that composes the ratio of the first and second derivatives of the waveform length in the non-linear scale, which is described to be less variant to the amplitude scaling of the EMG signal.
**AAC**	Average amplitude change	$\mathrm{AAC} = \frac{1}{N}\sum_{n=1}^{N-1}\lvert x_{n+1} - x_n\rvert$	AAC estimates the absolute difference between the surrounding EMG data segments represented as waveform length.
**EWL**	Enhanced wavelength	$\mathrm{EWL} = \sum_{n=2}^{N}\lvert (x_n - x_{n-1})^p\rvert$ $p = \begin{cases} 0{,}75, \text{if} \geq 0.2N \,\&\, n \leq 0.8N \\ 0.5, \text{otherwise} \end{cases}$	EWL (as EMAV) prioritizes the area within the middle segment of the wavelength to enhance the interpretation of the signal. p is the parameter of the enhanced wavelength feature.

**NSV**	Non-linear scaled value	$\mathrm{NSV} = \log_e\left(\sqrt{\frac{1}{N}\sum_{n=1}^{N}(\lvert\bar{x}\rvert - \lvert x_n\rvert^{1/3})^2}\right)$	NSV is tuned to disclose the non-linear deviation of the LMAV function in order to represent the low amplitude EMG signal rather than the high range.
**ZC**	Zero crossing	$\mathrm{ZC} = \sum_{n=1}^{N-1} \mathrm{zcf}(x_n)$ $\mathrm{zcf}(x_n) = \begin{cases} 1, \text{if } x_n x_{n+1} < 0 \\ 0, \text{otherwise} \end{cases}$	ZC counts the positive-to-negative ratio within a certain period of the signal length amplitude and stores the value as a feature. ZC measures the signal frequency change in the time domain and requires threshold values L to avoid false positives and isolate the signal noise.
**SSC**	Slope sing change	$\mathrm{SSC} = \sum_{n=2}^{N-1} \mathrm{sscf}[(x_n - x_{n-1})(x_n - x_{n+1})]$ $\mathrm{sscf}(x) = \begin{cases} 1, \text{if } x \geq \text{threshold} \\ 0, \text{otherwise} \end{cases}$	SSC returns the number of slope sign changes of the three proximate signal segments. The feature demands a threshold for optimizing statistical output and avoiding the background noise artifacts of the EMG.
**IRF**	Irregularity factor	$\mathrm{IRF} = \frac{\mathrm{ZC}}{\mathrm{SSC}}$	IRF as a function captures the ratio between the number of times when the signal crosses through zero value divided by a number of peaks (the number when the sign of signal slope changes).
**WAMP**	Willison amplitude	$\mathrm{WAMP} = \sum_{n=1}^{N-1} f(\lvert x_n - x_{n+1}\rvert)$ $f(x) = \begin{cases} 1, \text{ifx} \geq \text{threshhold} \\ 0, \text{otherwise} \end{cases}$	Alike to ZC, WAMP dissects frequency parameters in the time-domain spectrum. However, WAMP evaluates the amplitude difference of pair adjacent time-series segments of the signal that exceeds the threshold bounds in order to diminish myoelectrical noise. The given principle is used for monitoring the motor unit action potentials and assessing muscle contraction grade.
**MYOP**	Myopulse percentage rate	$\mathrm{MYOP} = \frac{1}{N}\sum_{n=1}^{N} f(\lvert x_n\rvert)$ $f(x) = \begin{cases} 1, \text{ifx} \geq \text{threshhold} \\ 0, \text{otherwise} \end{cases}$	MYOP is a dimensionless average parameter of the myopulse output in the EMG signal, in which absolute values if exceeding the pre-defined threshold are returned as one and stored as a feature.
**ER**	Multi-channel energy ratio of the EMG	$E_j = \sum_{n=1}^{N} x_n^2(ch_j); j = 2, \ldots, M-1, k = j + 1, \ldots M$ $\mathrm{ER}_{jk} = \frac{E_j E_1}{E_k^2}$	ER measures the ratio of the absolute energy distribution between the pairs of EMG channels. The energy ratio is normalized by the following channel (starting from the first) of the binary ratio evaluation.
**LER**	Log of multi-channel energy ratio	$\mathrm{LER} = \log(\mathrm{ER})$	LER is the log of the energy of each binary channel ratio.
**MER**	Mean energy ratio of the EMG	$\mathrm{MER} = \frac{1}{M}\sum_{j=1}^{M} \mathrm{ER}_j$	MER returns the average energy ratio between the EMG channels.
**MLER**	Log of mean energy ratio	$\mathrm{MLER} = \log(\mathrm{MER})$	MLER is the logarithmic version of the MER feature.

**MXR**	Max energy ratio of the EMG	$MXR = \max(ER)$	MXR is the max value in the absolute energy ratio between the EMG channels.
**LMXR**	Log of max energy ratio	$LMXR = \log(MXR)$	Like LER and MXR, the LMXR feature is the log version of the max energy ratio of the EMG.
**MHW**	Multiple hamming windows	$MHW = \sum_{n=1}^{N} (W_n x_n)^2$	MHW segments the myoelectrical signal into overlapped windows and computes the EMG signal's energy difference over these time series. W_n is the width of the Hamming window function.
**MTW**	Multiple trapezoidal windows	$MTW_k = \sum_{n=1}^{N-1} \left(W_{n-n_k} x_n^2\right), k = 1,2,3$	MTW operates similarly to MHW, but the window parameters have a trapezoidal shape. n_k is the starting point of the k-th trapezoid window.
**HIST**	Simple histogram of EMG		HIST disintegrates the EMG signal range into equally spaced numerical segments. Following a simple histogram design, we used four segments.
**SAHT**	Square root simple histogram		SAHT is the HIST equivalent using square root parameters of the segmented components.
**AR2-6**	Autoregressive coefficients from 2nd to 6th order	$x_n = -\sum_{P=1}^{P} a_p x_{n-p} + w_n;\ P = 2,3,4,5,6$	A predictive autoregressive model (AR) with a certain order (P) represents every EMG signal datapoint in a linear combination of previous temporal EMG samples (x_{n-p}) and white noise error term (w_n). High-dimensional return values of that function are aggregated in a feature vector (auto-regressive coefficients a_p).
**CCAR**	Cepstral coefficients derived from the AR model	$CCAR = c_p; c_1 = -a_1$<br>$c_p = -a_p - \sum_{l=1}^{p-1} \left(1 - \frac{l}{p}\right) a_p c_{p-l}$	CCARS is the inverse Fourier transform model magnitude of the power spectrum in the logarithmic scale of the signal. In analogy to AR, CCAR uses auto-regressive coefficients to convert them into a feature vector with the set order conditions ($1 \le l \le p$). In our settings, the order was set to 3.
**LPC2-6**	Linear predictive coefficients from 2nd to 6th order	$x_n = b_0 + b_1 x_n + b_2(x_n - 1) \ldots + b_6(x_n - 5)$<br>$LPC = [b_0, b_1, b_2 \ldots b_6]$	LPC is a transfer function of the AR prediction model in which linear predictive coefficients rely on the calculation of the gain function for each order cycle, not the recurrent autoregressive coefficients.
**LCARD**	Logarithmic cardinality of the EMG signal	The feature's threshold is set to 0.001.	In contrast to the original cardinality used in myoelectric pattern recognition, LCARD examines the number of unique values in the time-series set of the EMG signal in a non-linear fashion.
**PERC1**	Percentile type 1	$card\{x_n / x_n < PERC75\} = \frac{75N}{100}$	PERC1 is the converted 75th percentile signal distribution. In the following definition, the *card* is the number of initial values in the given EMG signal range.
**PERC2**	Percentile type 2	$card\{x_n / x_n < PERC50\} = \frac{50N}{100}$	50th percentile signal distribution was set as a feature (PERC2) during the empirical examination.
**SEN**	Sample entropy	$SEN(x, m, r) = -\ln\left(\frac{A^m(r)}{B^m(r)}\right)$	SEN evaluates the signal complexity independent of the length of the time-series events, which is practical and used in muscle onset detection. In the described definition m is the maximum epoch length, r is the tolerance parameter, $A^m(r)$ and $B^m(r)$ reflect the dimensions of m+1 and m.

**AEN**	Approximate entropy	$AEN_{(m,r,N)} = \lvert \Phi^{m}(r) - \Phi^{m+1}(r) \rvert$ $\phi^{m}(r) = (N-m+1)^{-1} \sum_{n=1}^{N-m+1} \log C_{n}^{m}(r)$ $C_{n}^{m}(r) = \frac{N_{n}^{m}}{N-m+1}$	AEN measures the signal model complexity. This entropy quantifies the amount of regularity and the unpredictability of fluctuations over time series in muscle fatigue detection and points to the trigger muscle events. $C_{n}^{m}(r)$ is the correlation sum. N_{n}^{m} is the number of data points with threshold conditions necessary for the computation of the $C_{n}^{m}(r)$.
**FEN**	Fuzzy entropy	$FEN(N,m,r) = \lim_{N\to+\infty} (\ln \Phi_{m} - \phi_{m+1})$ $\phi_{m} = 1/(N-m) \sum_{n=1}^{N-m} [1/(N-m-1)] \sum_{p=1,p\neq n}^{N-m} D_{n,p}^{m}$ $D_{n,p}^{m} = \exp\left(-\frac{(d_{n,p})^{m}}{r}\right)$	FEN measures quantify of time series regularity which extrapolates the EMG signal's complexity degree in terms of fuzziness. Φ_{m} is the value of the mean average similarity. N is the sample set, m shows the used dimensions of the data sample, D_{np}^{m} is a degree of similarity within two samples, and r is an amplitude of the exponential parameter of the D_{np}^{m}.
**PEN**	Permutation entropy	$H(n) = \sum_{\pi=1}^{n!} p(\pi) \ln(p(\pi))$	PEN is similar to FEN in detecting the relative occurrences of the various trends in the signal, but its distribution patterns in the entropy model are considered (returns sorted data structure with the ascending order in the multi-dimensional shape). In the definition, the new sequence of performed permutations and combinations of the n-dimensional feature vector is n! whereas π is the different permutation modus, and $p(\pi)$ references the probability statistics of n! within the whole time series.
**MASP**	Modified amplitude spectrum of the EMG signal	$MASP_{(m)} = \sum_{k=k_{m}}^{M} \frac{\lvert fft_{k} \rvert}{k_{m}}$	MASP groups the power frequency bins k into five equitable segments and concatenates the average values of every EMG channel into a single feature.
**MLASP**	Non-scale modified amplitude of the EMG signal	$MLASP_{(m)} = \sum_{k=k_{m}}^{M} \frac{\log \lvert fft_{k} \rvert}{k_{m}}$	MLASP is the non-scale feature of MASP in which five equal segments of frequency bins have been converted into log values.
**MNF**	Mean frequency	$MNF = \sum_{j=1}^{M} f_{j}P_{j} / \sum_{j=1}^{M} P_{j}$	MNF is the mean frequency domain value of the signal power spectrum. MNF can be expressed as the sum of multiplied quantities of the EMG power spectrum, and the frequency divided by the total value of the spectral intensity.
**MMNF**	Modified mean frequency	$MMNF = \sum_{j=1}^{M} f_{j}A_{j} / \sum_{j=1}^{M} A_{j}$	MMNF is the average frequency value obtained from the amplitude spectrum. In the following description, f_{j} is the frequency parameter of the signal's spectrum at the fixed frequency bin j.
**MDF**	Median frequency	$MDF = \frac{1}{2} \sum_{j=1}^{M} P_{j}$	MDF feature divides the spectrum of the signal into two parts with the same quantity amplitude.
**MMDF**	Modified median frequency	$MMDF = \frac{1}{2} \sum_{j=1}^{M} A_{j}$	MMDF operates the same as MDF, but instead of power spectrum density, it is based on the amplitude spectrum (A_{j}). MMDF divides the frequency spectrum into two intervals with the same amplitude.
**TTP, SM0**	Total power, zero spectral moment	$TTP = \sum_{j=1}^{M} P_{j}$	TTP concatenates the whole range of the EMG frequency-domain power spectrum into a single feature.

**MNPV**	Mean power value of the EMG signal	$\mathrm{MNP} = \sum_{j=1}^{M} P_j \,/\, M$	MNP is the average value of the aggregated power spectrum of the EMG signal.
**MDPV**	Median power value of the EMG signal	$\mathrm{MDP} = \mathrm{median}\,(P_j \,/\, M)$	MDP is the median power value of the EMG power spectrum.
**PKF**	Peak frequency	$\mathrm{PKF} = f_j,\ j = \max_j(P_j)$	PKF determines the EMG frequency which has the maximum signal power spectrum in a certain signal slot.
**FRT**	Frequency ratio	$\mathrm{FRT} = \sum_{j=LLC}^{ULC} P_j \,/ \sum_{j=LHC}^{UHC} P_j$	FRT is the correlation feature that is used for binary classification of the muscle state (relaxation and contraction). FRT is calculated by the division of the low and high-frequency bound components of the EMG signal as a variable. The high-low frequency bands and cutoff settings (UHC, LHC, ULC, and LLC) can be set empirically or assigned in relation to the mean frequency (MNF).
**PSR**	Power spectrum ratio	$\mathrm{PSR} = \frac{P_0}{P} = \sum_{j=f_0-n}^{f_0+n} P_j \,/ \sum_{j=-\infty}^{\infty} P_j$	PSR rates the values of the maximum EMG signal power parameter (PKF) versus the total frequency ratio of the energy slot (FRT). In the following equation, the f_0 is a PKF return value and n is an integral EMG signal limit which was empirically set to 20, whereas the energy of P was set to 10 and 500 Hz to reflect the typical range of muscle activity.
**LPSR**	Logarithmic power spectrum ratio	$\mathrm{LPSR} = \log\left(\frac{P_0}{P}\right)$	LPSR is PSR modification in which values are set to the logarithmic in order to decrease the size of the magnitude difference.
**SM1-3**	Spectral moments (1st, 2nd, and the 3rd)	$\mathrm{SM}_m = \sum_{j}^{M} (P_j f_j)^m,\ m = 1,2,3$	SM1-3 performs frequency-domain power spectral density evaluation of the EMG signal. High-order statistical spectral moment analysis discloses non-linear and non-Gaussian properties of the EMG signal and is used in muscle fatigue evaluation.
**SMN**	Spectral mean density	$\mathrm{SMN} = \frac{\sum_{j=1}^{M} f_j \mathrm{PSD}j}{\sum_{j=1}^{M} \mathrm{PSD}j}$	Keeping different statistical properties of power spectrum density (PSD) of the EMG signal in the discrete set or continuous function of frequency, SMD and SMN were used as alternative implementations of the MDF and MNF. SMD and SMN are arranged to describe the amount of PSD at a particular frequency during the observing mean and frequency density of the signal, in which the direct amplitude and frequency bin size ambiguity is foreclosed.
**SMD**	Spectral median density	$\mathrm{SMD} = \frac{1}{2}\sum_{j=1}^{M} \mathrm{PSD}_j$	
**FDD**	Fundamental frequency standard deviation	$\mathrm{FDD} = \mathrm{STD}(F_0)$	In the EMG practice, FDD observes the myoelectrical fundamental frequency standard deviation directly derived from the muscle signal and estimates its spectrum density information. F_0 is the fundamental frequency of muscle contraction.
**FRHT2,4**	The frequency histogram of the EMG		FRHT is a distribution of the signal's power spectrum over the divided frequency amplitude bins, in which each segment contains the percentage of signal power. Since histogram-related features have adjustable parameters, we set bins to 2 and 4.
**STFT**	Short-time Fourier transform	$\mathrm{STFT}(k, m) = \sum_{r=1}^{N-1} x\,(r) g(r-k) \epsilon^{-j2\pi mi/N}$	STFT transforms the EMG signal spectrum wave into various magnitude characteristics and evaluates non-stationary parameters in the time-series scale. In the given description, g is a window range function, whereas k and m reflect the signal's frequency and corresponding bins.

**EWT 4,6,8,10**	The energy of wavelet coefficient	$EWT = \sqrt{\frac{1}{K}\sum_{k=1}^{K} W_{J,k}^2}$	EWT accumulates the coefficients obtained from the discrete wavelet transform of energy in the initial signal. In the mathematical definition, K is the number of the jth layer decomposed coefficient level, and $W_{J,k}$ is the kth coefficient of the given layer decomposed coefficients. The Db4, 6, 8, and 10 wavelets and five-layer decomposition we set in the following feature.
**EWP 4,6,8,10**	The energy of the wavelet packet coefficient		EWP as an extended version of EWT encompasses wider signal ranges through each high and low-pass band and thereby has more signal contents inside of coefficients. Since EWP coefficients are massive, the median value parameter was used as a feature.
**ZCWC**	Zero crossing of the energy wavelet coefficients	$ZCWT = \sum_{j=1}^{K} u(-W_j W_{j+1})$	ZCWC performs zero-crossings (ZC) evaluation of the signal in the predetermined wavelet decomposition settings (in our study we used Db6); u is a unit-step function parameter.
**HHT**	Hilbert-Huang transform		HHT is used for measuring non-stationary events (such as muscle fatigue monitoring), it comprises the signal empirical mode decomposition and original Hilbert transforms spectral function. HHT is one of the most frequently used timescale features since it is able to extract useful features in a short-time EMG signal energy.
**SWT**	Stockwell transform		SWT is generated from the short-time Fourier transform and further wavelet decomposition that can adjust the window function settings during the signal analysis. Stockwell transform is used as a noise-gate feature in biomedical applications.
**FRFT**	Fractional Fourier transform		FRFT is the linear integral transform of the Fourier transform with the specific decomposition settings which return an equal number of the time-scale coefficients to the number of samples. It is shown that FRFT as a feature extraction method demonstrates efficient classification in gesture recognition.
**DWT**	Discrete wavelet transform		DWT function disintegrates the original signal into an approximation and itemizes obtained coefficients through low- and high-pass filters. The obtained coefficients are divided by next-level approximations which results in numerous components of lower resolution.
**WENT**	Wavelet entropy	$E_m = \frac{\sum_{n=1}^{N} \lvert C_m(n) \rvert^2}{\sum_{m=1}^{N} \sum_{n=1}^{N} \lvert C_m(n) \rvert^2}$ $WENT = -\sum_{n=1}^{N} E_m \log(E_m)$	WENT determines the degree of disorder that the signal values are possesses. This time-scale feature can provide useful information about the low-elemental complex processes tied to the initial EMG signal which is used in biomedical applications. In provided definition, m is the scale of the wavelet coefficients, E_m expresses the energy of each time sample n.
**SMAV**	Scaled mean absolute value	$SMAV = \frac{MAV_n}{\frac{\sum_{n=1}^{ch} MAV_n}{ch}}$	SMAV estimates the non-dimensional spatial relationships of EMG signals in between the channels, the results of which are stored as a space-domain feature. Spatial features eliminate the possible channel-specific excess signal intensity of the particular gesture by scaling the local MAV (in every single channel) by the average spatial mean value (obtained from all channels).
**MSMAV**	Modified scaled mean absolute value	$MSMAV = \frac{MAV_n}{\sqrt{SMMAV}}$	MSMAV is a SMAV function obtained using the root mean square of the mean of MAVs across all channels.

**CC-D**	Correlation coefficient (CC) of the normalized values using median value	$\mathrm{CCD} = \frac{\sum_{n=1}^{N} x_{ch}[n] x_{ch+1}[n]}{\mathrm{median}(\sum_{n=1}^{N} x_{ch}[n]^2)}$	The correlation coefficients between the individual channels' mean values track the muscle cross-talk in order to estimate the myoelectrical source of certain gestures. Due to required normalization, the output mean absolute values of that function are divided by their standard deviation.
**CC-S**	CC of the normalized values using square root value	$\mathrm{CCS} = \frac{\sum_{n=1}^{N} x_{ch}[n] x_{ch+1}[n]}{\mathrm{sqrt}(\sum_{n=1}^{N} x_{ch}[n]^2)}$	CC which are normalized with the median parameter of mean absolute value standard deviation.
**CC-R**	CC of the normalized values using root mean square value	$\mathrm{CCR} = \frac{\sum_{n=1}^{N} x_{ch}[n] x_{ch+1}[n]}{\mathrm{rms}\left(\sum_{n=1}^{N} x_{ch}[n]^2\right)}$	CC which are normalized with the root mean square parameter of mean absolute value standard deviation.
**RT4**	Mean absolute difference of the normalized values as a spatial ratio of four EMG channels	$\mathrm{RT4} = \frac{MAV_{(ch1)}}{MAV_{(ch2)}} + \frac{MAV_{(ch1)}}{MAV_{(ch3)}} + \frac{MAV_{(ch1)}}{MAV_{(ch4)}} + \frac{MAV_{(ch2)}}{MAV_{(ch3)}} + \frac{MAV_{(ch2)}}{MAV_{(ch4)}} + \frac{MAV_{(ch3)}}{MAV_{(ch4)}}$	Mean absolute difference of the absolute values between four EMG channels (RT4) as a metric of biomechanical relation between the muscles.
**FER-4**	Ratio of absolute mean values between flexors and extensors	$\mathrm{RT4} = \frac{MAV_{(ch1)} + MAV_{(ch2)}}{MAV_{(ch3)}} + \frac{MAV_{(ch1)} + MAV_{(ch2)}}{MAV_{(ch4)}} + \frac{MAV_{(ch3)}}{MAV_{(ch4)}}$	This hand-crafted feature examines the ratio of channels between flexors and extensors, normalized by the additional electrodes. Since each forearm and hand gesture has a specific biomechanical characteristic (and represents specific muscle pattern synergy), the certain antagonist's muscle behavior and its signal amplitude could specify pattern recognition.
**FRD, FR4**	Fractal dimension	$\mathrm{FRD}(k) = \frac{\left\{\left(\sum_{n=1}^{N/k} \lvert x(nk) - x((n-1)k) \rvert\right) \frac{N-1}{N}\right\}}{k}$	In terms of muscle behavior, the FRD feature designates the strength of muscle activity in which EMG parameters tend to have self-similarly during the scaling of the initial signal. k as a time-step was set to 4 in our study.
**DFA**	Detrended fluctuation analysis		DFA is a non-linear fractal algorithm that analyses the non-stationary characteristics of the EMG which is applicable in noisy and low-grade signal evaluation for pattern recognition.
**HFD**	Higuchi's fractal dimension		In biomedical applications, HFD evaluates muscle strength and the contraction grade. This fractal function measures the size and complexity of the EMG signal in the time-domain spectrum without fractal attractor reconstruction methods.

In a given data sample or segment, the EMG signal is represented by x_n, while N denotes the length of the EMG signal or window size. The amplitude spectrum of the signal in a particular frequency bin j, represented by A_j, is used to extract MMDF and other FD features. The power spectral density (PSD) is represented by SND. The frequency of the spectrum at frequency bin j is represented by F_j, while the EMG power spectrum at frequency bin j is represented by P_j. The length of the frequency bin is denoted by M.

Table S2. Feature sets comparison in hemiplegic hand gesture classification by single feature classification														
**Feature**	**CCR (%)**	**SD (%)**	**Feature**	**CCR (%)**	**SD (%)**	**Feature**	**CCR (%)**	**SD (%)**	**Feature**	**CCR (%)**	**SD (%)**	**Feature**	**CCR (%)**	**SD (%)**
IEMG	61.7143	11.1916	RMSV2	54.4286	13.2675	MYOP	51.7857	13.6351	AEN	35.6429	9.3322	EWT-6	59.3571	15.2521
AAV	30.3571	9.8104	LRMSV2	55.5000	15.1559	ER	21.5000	7.8964	FEN	30.4286	9.3941	EWT-8	63.0714	13.1220
MAV	54.5000	12.7474	RMSV3	53.5000	15.6128	LER	45.2857	14.2936	PEN	27.2143	9.0062	EWT-10	58.7857	12.1768
LMAV	55.4286	16.9311	LRMSV3	57.0000	14.8163	MER	21.2857	7.9927	MASP	60.5000	13.0195	EWP-4	30.5714	12.3543
MMAV1	54.4286	13.5747	RSM0	62.8571	11.8396	MLER	43.7143	14.9306	MLASP	60.4286	12.6974	EWP-6	33.2857	9.8022
MMAV2	54.0714	14.3668	RSD1	61.2143	15.2034	MXER	22.1429	8.9951	MNF	27.5714	10.5517	EWP-8	27.7857	9.0746
MMAV3	53.6429	12.6190	RSD2	59.6429	14.0447	LMXER	40.4286	12.4846	MMNF	23.9286	10.7623	EWP-10	27.9286	9.0291
MMAV4	53.2143	14.0813	MSR	55.7143	13.9573	MHW	62.2143	9.3763	MDF	28.7143	8.9652	ZCWC-6	32.7143	10.0082
MMAV5	56.2857	13.2480	ASM	54.0714	13.9667	MTW	58.6429	13.5562	MMDF	24.7857	11.0028	HHT	61.3571	11.5352
MMAV6	55.0714	14.9311	MAPN	54.7857	15.1627	AHT	35.3571	9.2144	TTP	48.6429	14.3826	SWT	31.0000	11.2238
MAVS	24.2143	8.3036	SD	53.8571	11.6386	SAHT	36.5714	9.2649	MNP	48.3571	14.4954	FRFT	52.5000	14.8993
EMAV	57.1429	14.3577	LOG	55.6429	12.7733	AR2	24.8571	11.3725	MND	55.1429	14.2886	DWT	60.0000	13.9941
SSI	61.0714	12.4497	ROG	57.5000	13.0952	AR3	33.3571	11.3078	PKF	29.0000	11.0199	WENT	55.2143	13.6154
LSSI	56.7857	11.7687	MDPV	55.7857	17.9240	AR4	31.4286	14.1771	FRT	25.5000	9.4289	SMAV	52.2143	13.2252
VAR	56.4286	13.1394	MPDV	58.4286	15.6090	AR5	33.8571	14.2720	PSR	26.4286	12.8620	MSMAV	57.7143	14.7010
LVAR	54.0714	13.4402	DASDV	55.2143	13.1534	AR6	33.4286	14.4607	LPSR	26.7857	11.2309	CC-D	57.0714	12.8216
HPM	26.3571	9.9832	MFL	58.4286	16.1605	CCAR	32.1429	13.7527	SM1	47.2143	16.9243	CC-S	29.3571	6.2497
HPC	28.4286	9.1923	WL	62.0714	13.3799	LPC2	25.3571	10.4217	SM2	51.5000	17.0577	CC-R	58.2143	11.5922
TM3	52.2857	14.7780	WLR	21.6429	10.2763	LPC3	30.4286	13.0655	SM3	52.0000	15.2313	RT-4	43.5000	11.7564
TM4	39.4286	11.4628	AAC	55.5000	16.0156	LPC4	35.1429	10.9665	SMN	45.8571	12.1905	FER-4	42.0714	10.5943
TM5	32.3571	9.2746	EWL	51.0000	15.9887	LPC5	32.4286	9.4924	SMD	47.6429	13.4708	FR4	63.9286	10.6176
MSKEW1	30.9286	12.9241	NSV	61.4286	14.6772	LPC6	31.6429	11.4527	FDD	22.9286	11.0887	DFA	57.2857	11.9255
MSKEW2	56.3571	17.6983	ZC	54.9286	19.1204	LCARD	59.0000	15.4176	FTHT2	32.5000	9.7577	HFD5	28.6429	10.5749
MSKEW3	35.1429	13.4947	SSC	51.2143	19.2107	PERC1	55.8571	13.6353	FTHT4	30.2857	9.2560			
KURT	30.8571	6.5701	IRF	50.5000	17.4449	PERC2	55.4286	12.8065	STFT	60.7857	11.2822			
MKURT	39.5000	12.4530	WAMP	56.0000	17.5763	SEN	26.3571	9.5613	EWT-4	58.2857	14.0573			

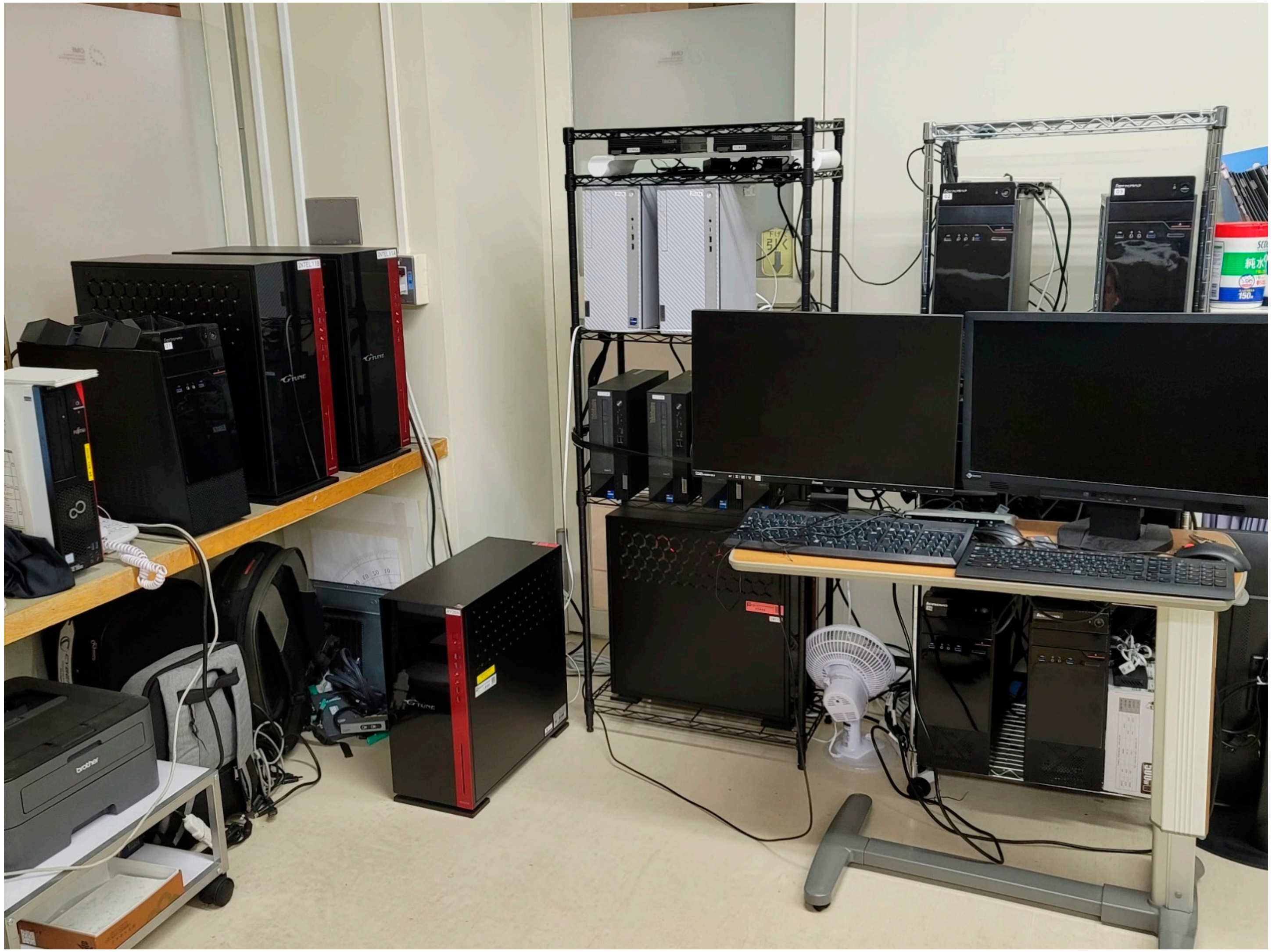

Figure S1. Custom-made consumer-type parallel computing cluster or "3C cluster". The SVM cross-validation was performed using the EMG dataset of 19 acute stroke patients with various grades of upper extremity paresis specified.

Table S3. Feature set comparison in hemiplegic hand gesture prediction (*n* = 100 and equals to the number of 10-fold cross-validations) using SBFS5.

Feature set	CCR (%)	SD (%)	DF	*p*-Value
SBFS5	69.5000	12.5929	---	---
MFS1	60.5000	13.4478	198	2.1284×10^{-6}****
MFS2	47.7857	16.4862	198	1.0926×10^{-20}****
MFS3	57.5714	16.2064	198	2.4360×10^{-8}****
MFS4	57.5000	15.1395	198	5.6569×10^{-9}****
MFS5	60.4286	12.5340	198	7.7153×10^{-7}****
MFS6	55.1429	14.3606	198	1.9001×10^{-12}****
MFS7	53.2143	13.7105	198	9.4246×10^{-16}****
MFS8	55.5714	13.3372	198	1.2035×10^{-12}****

**** shows significance with $p < 0.0001$. SBFS—semi-brute-force search, MFSs—multi-domain feature sets, SD—standard deviation, CCR—mean correct classification rate, and DF—degree of freedom during the unpaired *t*-test.
